# Reducing prescribing cascades

**DOI:** 10.4102/phcfm.v17i1.4929

**Published:** 2025-03-31

**Authors:** Aaron M. Tejani, Thomas L. Perry

**Affiliations:** 1 Department of Anaesthesiology, Pharmacology and Therapeutics, Faculty of Medicine, University of British Columbia, Vancouver, Canada

**Keywords:** prescribing cascade, polypharmacy, adverse drug events, deprescribing

## Abstract

Prescribing cascades contribute to the increasing prevalence of polypharmacy and its associated risks, where a drug-induced adverse event is misinterpreted as a new condition and treated with additional medications. Notable cascades include the use of anticholinergics leading to cognitive impairment, dyspepsia or constipation, which then prompt prescriptions for dementia medications, proton pump inhibitors or laxatives, respectively. Similarly, calcium channel blockers and gabapentinoids often induce oedema, resulting in unnecessary diuretic use. Strategies for prevention include careful review of adverse effects, deprescribing where appropriate and clinician education to improve symptom interpretation and prescribing practices. Recognising these cascades can mitigate unnecessary interventions and improve patient outcomes.

## Case vignette

A 70-year-old woman is diagnosed with chronic obstructive pulmonary disease (COPD), obstructive sleep apnoea, left ventricular dysfunction with outflow obstruction, hypertension, prior pulmonary embolism, insomnia, anxiety, depression, mild cognitive impairment and unexplained laryngitis. She takes 14 prescription drugs and 4 supplements (see the following chart), but would prefer fewer. Her primary care nurse practitioner consults the family physician for a comprehensive medication review.

**Table:** 

**Cardiac and blood pressure**	
1.	Spironolactone 25 mg/day morning
2.	Diltiazem extended-release 180 mg/day morning
3.	Carvedilol 25 mg/day morning
4.	Furosemide 40 mg/day morning
5.	Rosuvastatin 10 mg/day at night
**Prevent venous thromboembolism**	
6.	Rivaroxaban 20 mg/day evening
**Suspected acid reflux** (no gastrointestinal [GI] bleed)	
7.	Pantoprazole 40 mg/day morning
**COPD**	
8.	Tiotropium 2.5 mcg (2 inhalations) morning
9.	Budesonide 100 mcg/formoterol 6 mcg (2 puffs) morning
10.	Salbutamol 100 mcg (2 puffs) as needed
**Anxiety/depression/sleep**	
11.	Venlafaxine 300 mg/day morning
12.	Mirtazapine 30 mg/day at night
13.	Clonazepam 0.25 mg/day at night
14.	Melatonin 3 mg/day at night
15–18.	Iron, calcium, vitamin C, vitamin D

COPD, chronic obstructive pulmonary disease.

## Are prescribing cascades hiding in plain sight?

Once unimaginable, intimidating drug lists are now common.^1^ Mitigation may be easier if prescribers and dispensing pharmacists recognise the potential for ‘prescribing cascades’. Coined by two geriatricians in 1995:^2^

[*A*] prescribing cascade begins when a drug is prescribed, an adverse drug event occurs that is misinterpreted as a new medical condition, and a subsequent drug is prescribed to treat this drug-induced adverse event.^3^

Sequelae also include over-the-counter medicines or medical devices (e.g., cardiac pacemaker insertion).

Potential *prescribing cascades* in this vignette include:

**Table:** 

Drug	Common adverse effect	Possible cascade prescriptions
Diltiazem	Peripheral oedema	Furosemide for oedema mistaken as volume overload or right heart failure
Tiotropium	Anticholinergic: hoarseness/laryngitis	Pantoprazole to reduce stomach acid (but not reflux)
Budesonide	Corticosteroid: Candidiasis/hoarseness	Pantoprazole
Mirtazapine	Anticholinergic: impaired stomach emptying; hoarseness/laryngitis	Pantoprazole
Venlafaxine	Nausea, ‘indigestion’, gastric upset	Pantoprazole
Venlafaxine	Insomnia, agitation, anxiety	Clonazepam, melatonin, mirtazapine
Venlafaxine	Tachycardia/palpitations	Increased dose of bisoprolol
Pantoprazole	Impairment of iron absorption	Iron supplement
Iron (FeSO_4_)	Nausea, indigestion	Pantoprazole

Prescriptions added to counter one or more drug effects could induce falls from oversedation, ‘mild cognitive disorder’ or other long-term anticholinergic (antimuscarinic) effects, or adverse effects of a proton pump inhibitor (PPI). Given this woman’s interest in deprescribing, the family physician also questioned other drugs in her list.

## What is known about prescribing cascades?

While global knowledge on prescribing cascades is increasing,^4^ literature from lower-income countries and the recognition of prescribing cascades in these resource-limited regions are still low.^5^ Published studies of prescribing cascades focus on several drug classes,^6^ with selected examples are discussed next.

## Seven prevalent examples

### Anticholinergic drugs → cognitive dysfunction → drugs for dementia

Anticholinergics (e.g., tricyclic antidepressants, cyclobenzaprine, mirtazapine, quetiapine and oxybutynin)^7^ block acetylcholinergic neurotransmission in the brain, impairing cognition and memory even in the presence of acetylcholinesterase inhibitors (AChE-I: donepezil, galantamine, rivastigmine).^8,9^ Cognitive decline may be perceived as a new condition or worsening dementia, and can lead to new prescriptions or increased doses of AChE-I.^10,11^

### Drugs for dementia → incontinence → anticholinergics

Conversely, AChE-I can cause urinary or faecal incontinence that may ‘cascade’ to the prescription of an anticholinergic. Two studies found increased use of antimuscarinic bladder drugs (e.g., oxybutynin) after the prescription of cholinesterase inhibitors for dementia.^12^ Bradycardia or syncope (muscarinic) or muscle cramps (nicotinic) are other cholinergic effects that may precipitate new treatments.^13,14,15^

### Anticholinergics → dyspepsia/reflux (gastroesophageal reflux disease — ‘GERD’) → Proton pump inhibitor

Dyspepsia or heartburn because of delayed gastric emptying can be mistaken for spontaneous gastrointestinal reflux or labelled loosely as ‘GERD’ (gastroesophageal reflux disease). This association was suggested as a possible cascade in a study evaluating longstanding (‘legacy’) prescriptions of PPIs.^16^ In a United States of America (US) study of 248 nursing home residents, the likelihood of receiving a PPI increased with anticholinergic burden.^17^ Similarly, a large Canadian cohort study of seniors with dementia suggested that anticholinergics increased PPI dispensing ‘consistent with a prescribing cascade’.^18^

### Anticholinergics → constipation → laxatives

Drug-induced constipation is well recognised, an association confirmed by a 2021 systematic review.^19^ Among Italian nursing home residents, tricyclics increased laxative use (odds ratio [OR] 2.98, 95% confidence interval [CI] 1.31–6.77), as did other antidepressants, especially mirtazapine (OR 1.37, 95% CI 1.09–1.71).^20^

### Calcium channel blockers/gabapentin/pregabalin → oedema → diuretics

Dihydropyridine calcium channel blockers (CCB) frequently cause dose-dependent oedema, affecting up to 30% of older patients.^21,22^ Two recent cohort studies found that furosemide prescriptions increased in people taking CCBs, compared with other antihypertensives.^23,24^ Reducing or stopping a CCB can be preferable to adding furosemide, given its multiple adverse effects.

Gabapentin and pregabalin also cause dose-dependent peripheral oedema. In chronic pain, this affects up to 9% of people taking gabapentin and 10% for pregabalin (up to 4-fold vs. placebo).^25,26,27^ A large Canadian cohort study from 2011 to 2019 found increased loop diuretic prescriptions following initiation of gabapentin/pregabalin for new onset low back pain in older adults (hazard ratio [HR] 1.44, 95% CI 1.23–1.70; absolute risk increase 0.7%).^28^ Both may be associated with an inappropriate diagnosis of heart failure.^29^

### Drug-induced movement disorders → antiparkinsonian drugs

Most antipsychotics, some antidepressants, and the anti-emetics metoclopramide and prochlorperazine block dopamine receptors or cause movement disorders by other mechanisms. Such adverse events can be mistaken for Parkinson’s disease.^30^ While a Canadian study found these prescribing cascades unusual,^12^ others see more reason for concern. Prescriptions for newer as well as older antipsychotics, antidepressants and metoclopramide have been associated with increased subsequent prescriptions of L-dopa/carbidopa and other anti-Parkinsonian drugs.^3,30,31,32,33,34^

### Drug-induced hypertension → antihypertensive drugs

About 15% of American adults (19% of adults with hypertension) take a drug that can raise blood pressure.^35^ Antidepressants (8.7% of adults) and prescription non-steroidal anti-inflammatory drugs (NSAIDs) (6.5% of adults) were the most frequent potential candidates for an under-recognised prescribing cascade.

## Reducing prescribing cascades

Preventing, detecting and reversing prescribing cascades are not easy.^6,36,37,38^ Recognising and intercepting cascades still require knowledge and expert medication review, including attention to known cascades.^39^ A rural medical reviewer who reviewed this Letter wrote:

‘The problem is largely our mindset of reflexively treating new symptoms with medications, without first thinking of drug-induced side effects in patients already taking many. We need to think more, before taking the easy option of reaching for the prescription pad.’

## Conclusions

- Prescribing cascades cause avoidable polypharmacy and harms.
- Prevent them by careful indication-based prescribing and screening for cascades during medication reviews. Utilise expert pharmacist or medical consultation when available.
- Start by familiarisation with cascades involving drugs common in primary care; reduce doses if deprescribing seems too radical.
- Identifying a prescribing cascade is a teachable moment: use it.
